# Healthcare workers’ and caregivers’ knowledge, perceptions and experiences of the school-based human papillomavirus (HPV) immunization program: A qualitative study in eThekwini District of the KwaZulu-Natal Province, South Africa

**DOI:** 10.1080/21645515.2026.2613571

**Published:** 2026-01-13

**Authors:** Phelele Bhengu, Charles Shey Wiysonge, Vuyolwethu Magasana, Sara Cooper, Mosa Moshabela, Patrick de Marie C. Katoto, Duduzile Ndwandwe, Muki Shehu Shey

**Affiliations:** a Department of Medicine & CIDRI-Africa, Faculty of Health Sciences, University of Cape Town, Cape Town, South Africa; b Cochrane South Africa, South African Medical Research Council, Cape Town, South Africa; c Division of Epidemiology and Biostatistics, Department of Global Health, Stellenbosch University, Cape Town, South Africa; d HIV and Other Infectious Diseases Research Unit, South African Medical Research Council, Durban, South Africa; e Vice-Chancellor’s Office, University of Cape Town, Cape Town, South Africa; f Centre for Tropical Diseases and Global Health, Catholic University of Bukavu, Bukavu, Democratic Republic of Congo; g Centre for General Medicine and Global Health, Department of Medicine, University of Cape Town, Cape Town, South Africa

**Keywords:** Human papillomavirus, cervical cancer, vaccine confidence, vaccine safety, vaccination, Africa

## Abstract

The introduction of human papillomavirus (HPV) vaccination programs is a significant achievement in preventing cervical cancer and other HPV-related illnesses. This study aimed to explore healthcare workers (HCWs) and caregivers (CGs) knowledge, perceptions and experiences surrounding the school-based HPV immunization program in eThekwini District, KwaZulu-Natal Province, South Africa. CGs refer to parents or other individuals responsible for making vaccination decisions for eligible girls. A qualitative study design was employed that incorporated in-depth, semi-structured interviews with 20 CGs and 20 HCWs from different areas of eThekwini District who are involved in a school-based HPV immunization program. The study was informed by the World Health Organization’s Measuring Behavioral and Social Drivers of Vaccination (BeSD) approach. The BeSD resources include qualitative tools for conducting in-depth interviews that informed our interview guide. Thematic analysis was used to analyze interviews that were recorded, transcribed, and translated. HCWs indicated a thorough understanding of HPV and the vaccine’s benefits, but CGs’ knowledge varied, with some having misconceptions about the vaccine. HCWs largely praised the school-based HPV immunization program initiative but noted practical difficulties. CGs’ perceptions varied from supportive to doubtful, depending on the information they received. Common impediments highlighted included a lack of information, cultural and religious beliefs and communication breakdowns. The study established that factors such as culture, information, and interpersonal experiences influence the knowledge, acceptance, and uptake of the HPV vaccine. The findings are anticipated to guide development of tailored interventions to increase HPV vaccination coverage in South Africa.

## Introduction

Human papillomavirus (HPV) is one of the most common sexually transmitted diseases (STIs) globally.^1^ HPV is known to cause various cancers, including cervical cancer, which all have a significant global burden. There are approximately 630,000 cervical cancer cases reported every year, with cervical cancers making up 84%, making it the fourth most common cancer in women.^1^ Recent studies confirm that around 84% of the cases occur in low- and middle-income countries (LMICs) partly due to the high prevalence of HIV, which can make it challenging to effectively manage HPV immunologically.^2^ Existing HPV vaccines have demonstrated exceptional safety and effectiveness, particularly against HPV types 16 and 18, which are the main strains associated with HPV-related cancers.^3–5^

In South Africa, a school-based HPV vaccination program was introduced in 2014 to administer the vaccine to Grade 4 girls, typically aged nine years and above, using a two-dose regimen spaced six months apart.^6,7^ By 2018, approximately 1.3 million girls aged 9–14 y had received the vaccine, marking an important public health achievement.^8^ However, significant regional disparities persist. While national first-dose coverage exceeds 80%, some sub-districts in KwaZulu-Natal report rates as low as 40%; indicating substantial challenges in achieving equitable vaccine access and uptake.^9^

The effectiveness of such programs depends not only on vaccine supply but also on the knowledge, attitudes, and perceptions of key stakeholders, particularly healthcare workers (HCWs) and caregivers (CGs). The role of HCWs in this program is multifaceted, encompassing vaccine administration, education on vaccine benefits and risks, and managing adverse reactions.^10^ Research indicates that recommendations from HCWs are a strong predictor of vaccine uptake, emphasizing the importance of their commitment and advocacy in the success of the vaccination program.^11^ Likewise CGs, defined as parents or guardians responsible for children’s health decisions, substantially influence vaccine acceptance. Evidence from South Africa and other sub-Saharan African contexts identifies recurring barriers such as misinformation, cultural taboos surrounding sexual health, and limited awareness of HPV’s link to cervical cancer.^12,13^ These factors collectively contribute to vaccine hesitancy and suboptimal coverage in high-burden communities.

Despite the centrality of stakeholder engagement, few qualitative studies have examined the lived experiences of HCWs and CGs in South Africa’s school-based HPV immunization initiative, particularly in HIV and HPV high-prevalence settings such as KwaZulu-Natal Province. This study addresses this gap by exploring HCWs’ and CGs’ perspectives within the eThekwini District, applying the WHO Behavioural and Social Drivers of Vaccination (BeSD) framework to examine cognitive, social, motivational, and practical influences on vaccine acceptance and uptake.^14^ Findings aim to inform context-specific, culturally responsive strategies to enhance HPV vaccination coverage and advance cervical cancer prevention in South Africa and similar LMIC contexts.

### Conceptual framework

The study was informed by the BeSD approach developed by the WHO.^14^ The BeSD tools for childhood vaccination include a quantitative survey and detailed qualitative interview guides, which we modified and validated for use with regards to HPV vaccination in South Africa.

The WHO BeSD conceptual framework seeks to comprehend and address the behavioral and social factors that influence vaccination. This approach acknowledges that individuals’ health behaviors are influenced by a complex interplay of social, environmental, economic, and cultural factors. Specifically, the framework highlights 4 key drivers of vaccination. The first domain – “Thinking and feeling” – includes the cognitive and emotional responses toward vaccines. The second domain – “Social processes” - consists of the social norms and recommendations on vaccines. The third domain – “Motivation” – includes the willingness and hesitancy toward vaccination. Finally, the fourth domain – “Practical issues” – includes the practical barriers faced when getting vaccinated.^14^ Ultimately, the framework promotes a multi-sectoral, multi-factorial and comprehensive strategy for understanding, and in turn addressing, the complex drivers of vaccination.

## Methods

This qualitative study was conducted in eThekwini District, KwaZulu-Natal, South Africa, between June and August 2022 using semi-structured interviews (n = 40) for data collection. The research focused on the district’s school-based HPV immunization program and involved HCWs (n = 20) and CGs (n = 20) from the communities of Chatsworth, Embo, Umlazi, and Wentworth (Table 1). The research utilized a phenomenological research design with the intention of capturing a comprehensive understanding of the experiences and perceptions of these critical stakeholders.^15^ This approach aligns with contemporary qualitative methods in healthcare research, emphasizing the importance of subjective insights in understanding complex healthcare issues and interventions.^16^

**Table 1.** Characteristics of participants (N = 40).

Participants	Chatsworth	10 (5 HCWs, 5 CGs)
	Embo	10 (5 HCWs, 5 CGs)
	Umlazi	11 (6 HCWs, 5 CGs)
	Wentworth	9 (4 HCWs, 5 CGs)
Age (years) for CGs	<40	12
	40–49	4
	50–59	1
	60–70	3
	>70	0
Gender	Females	20 HCWs, 18CGs
	Males	2 CGs
HCWs job positions	School health nurses	19
	Private clinic nurse	1

### Participant recruitment

For this study, we used a recruitment method that considered the challenges of representative sampling, given the diverse ethnic and socio-economic backgrounds of South African HCWs and CGs. We thus used a maximum variation purposive sampling approach to ensure that participants came from diverse socio-economic situations, personal backgrounds and residential settings.^17^ Participants were selected to ensure diversity in factors such as income level, education, employment status, and residential setting (urban, peri-urban, and rural). This strategy allowed us to identify common patterns across diverse experiences while also exploring context-specific influences.

### Study setting and context

Recruitment activities were held in four communities within eThekwini District: Chatsworth, Embo, Umlazi, and Wentworth. These communities were purposively selected to represent varying levels of healthcare service organization, resource availability, and levels of infrastructural development across communities. Embo, a small rural village in KwaZulu-Natal, represents the province’s least-developed communities, with high levels of unemployment and a majority of lower-middle-income people.^18^ Like other rural areas in the eThekwini District, Embo depends on sugar cane and maize. While Chatsworth and Umlazi are urban townships with specific socio-economic concerns, Wentworth is a peri-urban area which faces similar unemployment and infrastructure issues as the townships but with different demographic characteristics.^18^

### Recruitment process

Recruitment occurred in two phases over a four-week period. During the initial contact phase (weeks 1–2), research assistants visited public healthcare facilities, local schools, clinics, and hospitals in the four communities to distribute information brochures and introduce the study to potential participants. Information sessions were held with HCWs during staff meetings and with caregivers during school parent meetings or at clinic waiting areas. During the follow-up phase (weeks 3–4), researchers returned to schedule individual meetings with those who expressed interest in participating. Information brochures were provided 7–14 d before scheduled interviews to allow participants time to review study information, consult with family members if desired, and formulate questions. This staggered approach helped minimize coercion and ensured informed voluntary participation.

### Data collection process

Trained research assistants interviewed HCWs and CGs at Chatsworth, Embo, Umlazi, and Wentworth healthcare facilities, local schools, clinics and hospitals (see Table 1 for demographic details). The semi-structured interview questions were informed or guided by the BeSD conceptual framework to explore the four primary drivers: Thinking & Feeling, Social Processes, Motivation, and Practical Issues. Interviews lasted on average 20 minutes and were conducted in English or IsiZulu, depending on the participant’s preference. All interviews were audio-recorded with permission and were transcribed verbatim for data accuracy. Ensuring confidentiality and privacy, all personally identifiable information were carefully redacted from transcripts. Participants provided written, informed consent prior to their interviews and were given information leaflets.

### Data saturation

Data saturation was assessed using the approach recommended by Braun and Clarke, whereby the research team met after every 5 interviews to review emerging codes and themes.^19^ Saturation was determined to be reached when: (1) no new codes emerged from three consecutive interviews in each participant group, (2) existing themes were consistently reinforced without new dimensions emerging, and (3) the research team achieved consensus that sufficient depth and breadth of understanding had been attained. Data saturation occurred after 18 HCW interviews and 17 CG interviews; however, we completed all 20 scheduled interviews in each group to honor commitments made to participants and ensure sufficient data richness.

### Data analysis

Data were analyzed using thematic analysis based on inductive methodology, which is a recursive process with constant back and forth between thematic analysis phases, as defined by Braun and Clarke.^19^ This allowed the data to ‘speak for itself,’ which means that the constructs discovered and assessed as final themes were intrinsic, or ‘grounded’ in the data.^19^ Two researchers (Phelele Bhengu and Vuyolwethu Magasana) independently coded the first ten transcripts to enhance uniformity and develop an initial coding framework. After comparing their preliminary frameworks, it became apparent that a standard, comprehensive, and coherent coding framework was necessary. The coding was informed by the BeSD conceptual framework, with the four domains (Thinking and Feeling, Social Processes, Motivation, and Practical Issues) contributing to the generation of themes. For each theme, we considered the issue’s significance as well as the subjective, socio-cultural, and relational contexts in which it existed. Further discussions between the two researchers were then undertaken to check if these themes ‘fitted’ with the coded extracts and overall dataset, to refine the specifics of each theme, to understand the relationships between the themes, and to contextualize these themes within other relevant topics emerging from the data. The coding framework was then presented to the broader research team for further discussion, clarification, and refinement before proceeding with additional analyses. Regular meetings throughout the analysis process ensured consistency and rigor in theme development.

### Ethical considerations

Ethical approval was obtained from the University of Cape Town Faculty of Health Sciences Human Research Ethics Committee (HREC Reference: 286/2021). All participants provided written informed consent after receiving thorough explanations of the study purpose, procedures, risks, and benefits in their preferred language (English or isiZulu). Given that half of our participants were HCWs, we implemented specific measures to address potential ethical concerns. To mitigate power dynamics between researchers and HCW participants, all HCW interviews were conducted as per appointment and during non-working hours when possible. We explicitly emphasized that participation was entirely voluntary, would not affect their employment status or professional standing, and that their employers or supervisors would not have access to individual responses.

### Confidentiality and data security

To ensure participant anonymity, all data were de-identified immediately upon transcription, with only participant codes (HCW1-20, CG1-20) and community identifiers used in analysis. Audio recordings were stored on encrypted, password-protected devices accessible only to the core research team and will be destroyed five years after study completion in accordance with institutional policies. All transcripts and analytical documents are stored on secure, password-protected servers. Only aggregate findings are reported in publications and presentations, with no individual or facility identifiers disclosed. Quotations used in this manuscript have been carefully selected and edited where necessary to remove any potentially identifying details while preserving the essence of participants’ statements.

### Informed consent process

The informed consent process included a detailed explanation of the study, opportunities for participants to ask questions, and assurance that they could withdraw at any time without penalty. For participants with limited literacy, research assistants read the consent forms aloud and answered questions before obtaining written consent (signature).

## Results

### Participant characteristics

The 40 study participants included 20 HCWs and 20 CGs from four communities in eThekwini District (Table 1). Among HCWs, 19 were school health nurses employed by the Provincial Department of Health and 1 was a nurse working in a private clinic. All HCW participants were female. Caregivers ranged in age from under 40 to 70 y, with the majority (12/20, 60%) under 40 years old. Most caregivers were female (18/20, 90%), with 2 male participants. Participants were recruited from both urban areas (Chatsworth, Umlazi, Wentworth) and a rural area (Embo), providing diverse socio-economic and geographic perspectives. Table 1 presents the detailed demographic characteristics of study participants.

### Theme 1: knowledge and awareness of HPV vaccination program (BeSD domain: Thinking and Feeling)

For this study, we distinguished between ‘knowledge’ and ‘awareness’ as follows: *Knowledge* refers to accurate understanding of factual information about HPV, the vaccine, and its purpose (e.g., knowing that HPV causes cervical cancer, understanding the two-dose schedule, and recognizing that vaccination is recommended before sexual debut). *Awareness* refers to having heard of or being familiar with the existence of HPV or HPV vaccination programs without necessarily possessing detailed or accurate information. These distinctions guided our coding and interpretation of participants’ responses.

Knowledge and awareness of HPV and the HPV vaccination program are crucial for improving public health outcomes, especially in the school-based HPV immunization program.

This theme aligns with the BeSD framework’s *Thinking & Feeling* domain, which encompasses the cognitive appraisals and emotional responses toward vaccines. Our findings revealed distinct patterns in how HCWs and CGs understood and felt about HPV vaccination.

### Healthcare workers’ knowledge

HCWs demonstrated strong foundational knowledge about HPV, its relationship to cervical cancer, and the purpose of the school-based vaccination program. They understood their role as primary sources of health information, as one HCW explained:

So, we basically go out to the schools to guide the HPV campaign where we do health education about HPV, what its all about, the benefits of it and we issue the consents forms for the parents to sign.

(HCW17 - Wentworth1)

### Caregivers’ knowledge

In contrast to HCWs, CGs’ knowledge levels varied considerably, ranging from comprehensive understanding to near-complete ignorance. This variation appeared to be influenced by their access to healthcare information, educational background, and engagement with health systems. Some CGs demonstrated complete lack of awareness, suggesting significant gaps in information dissemination:

Mmm, not much. I havent actually heard or maybe I wasnt paying much attention when they were talking about it.

(CG3 - Chatsworth)

Conversely, other CGs showed good understanding and positive perceptions, indicating successful information reach in some cases:

Okay, actually my last born had taken the vaccine … I think its a good thing because it helps with the prevention of cervical cancer.

(CG16 - Wentworth)

### Urban-rural differences

Systematic differences emerged between urban and rural participants. Caregivers from Embo (rural area) demonstrated lower baseline knowledge about HPV compared to their urban counterparts. Specifically, 3 out of 5 rural CGs had not heard of school-based HPV vaccination program, compared to only 1 out of 15 CGs in urban areas (Chatsworth, Umlazi, and Wentworth). However, rural participants expressed higher trust in HCW recommendations once information was provided. Urban participants showed more diverse information sources, including social media, community networks, and previous healthcare encounters, but this also meant greater exposure to misinformation.

### Impact of misinformation

Both HCWs and CGs identified misinformation as a significant barrier shaping community perceptions of HPV vaccination. One HCW pointedly highlighted this challenge:

One major challenge is misinformation. There are a lot of myths and fears about the vaccine in the community.

(HCW9 - Embo1)

Despite these challenges, when effective communication occurred, it led to informed decision-making. A CG from Umlazi remarked:

I heard about it at the school meeting. They explained it well … I decided its important for my child.

(CG10 - Umlazi)

### Theme 2: culture and beliefs on school HPV vaccination (BeSD domain: Social Processes)

This theme aligns with the BeSD framework’s *Social Processes* domain, which examines how social and cultural norms, beliefs, and community values influence vaccination behaviors. Our findings revealed that cultural factors significantly shaped vaccine acceptance and hesitancy in eThekwini District.

### Cultural taboos around sexual health

HCWs observed that cultural hesitancy surrounding sexual health discussions constituted a major barrier to vaccine acceptance. In many communities within eThekwini, particularly in rural and traditional settings, topics related to sexuality are considered inappropriate for discussion, especially with children. One HCW explained:

In our community, talking about anything related to sexual health is often taboo. This makes discussing HPV vaccine a challenge.

(HCW8 - Embo3)

Similarly, these sentiments were further reinforced by another participant, noting how parents’ discomfort with sexuality-related topics created resistance. Similarly, these sentiments were further reinforced by another participant,

Some parents are hesitant because they think talking about HPV leads to discussions on sexuality, which theyre not comfortable with.

(HCW20 - Wentworth4)

### Language and cultural accessibility

An important cultural and practical barrier emerged regarding the language of vaccine information materials. Participants expressed concern that consent forms and educational materials provided only in English created barriers for isiZulu-speaking communities:

Sometimes it happens that the consent forms we have received are written in English and that pose a challenge most of the times we work with … schools so the parents will not understand them and you will find out that they do not completely fill out the form because they do not adequately understand the language. So, it would be great to preferably have isiZulu consent forms when working in Zulu schools.

(HCW14 - Embo2)

### Community skepticism and alternative belief systems

CGs’ perspectives revealed broader community skepticism toward modern medical interventions, rooted in preference for traditional medicine and long-held cultural beliefs. One participant observed:

In our circle, vaccines are sometimes seen as unnecessary. Its a battle against long-held beliefs.

(CG18 - Wentworth)

Another CG’s experience illustrated the tension between traditional and biomedical approaches:

I come from a background where traditional medicine is preferred. It was a big decision to go for the vaccine.

(CG5 - Chatsworth)

This skepticism was not merely about HPV vaccination but reflected broader distrust of vaccines and modern medicine. One CG articulated this general vaccine skepticism:

In our community, not everyone is convinced about vaccines. Theres a lot of scepticism and fear.

(CG9 - Umlazi)

Despite these cultural challenges, participants identified potential solutions. One HCW recommended cultural integration in health promotion, while a CG emphasized the value of community-based education:

Having someone from our community explain it in a way we understand made a big difference.

(CG11 - Umlazi)

Additionally, addressing the stigma surrounding sexual health discussions was identified as crucial:

Education should start early, breaking the taboo around these topics. (CG2 -Chatsworth)

### Theme 3: influence of healthcare workers in school vaccination programs (BeSD domain: Social Processes & Motivation)

This theme intersects the BeSD framework’s *Social Processes* and *Motivation* domains, demonstrating how trusted social relationships with HCWs influence individuals’ willingness to vaccinate. The findings established that HCWs and CGs serve as pivotal sources of information and trust within eThekwini communities.

### HCWs’ understanding of their influence

HCWs possess a profound understanding of their significant impact in endorsing and encouraging vaccine acceptance. They recognized that their professional recommendations carried substantial weight in communities:

As a nurse, I know parents look up to us for advice. When we speak positively about the HPV vaccine, it makes a big difference.

(HCW6 - Umlazi6)

HCWs understood their role extended beyond mere vaccine administration to education and reassurance:

Our role isnt just to administer the vaccine but to educate and reassure. Our words carry weight with the community. (HCW11 - Embo3)

### Caregivers’ trust in HCW recommendations

CGs consistently demonstrated that trust in HCWs was a decisive factor in their vaccine decision-making.

Even initially hesitant CGs were persuaded when HCWs provided clear information and addressed concerns:

I was hesitant, but the nurse explained everything and addressed my concerns. It helped me decide.

(CG6 - Chatsworth)

### Theme 4: system-level barriers to HPV vaccine uptake (BeSD domain: Practical Issues)

This theme aligns with the BeSD framework’s Practical Issues domain, which encompasses the tangible, logistical barriers that prevent vaccination even when individuals are motivated to be vaccinated. Understanding these system-level challenges is crucial for improving HPV vaccine coverage in eThekwini District.

### Accessibility and availability

Participants raised important concerns about equitable access to HPV vaccines. One HCW emphasized that vaccines should be accessible to all children, not just those reached through school programs:

I think it should be available [to every child]. Because if the vaccine is given to one child, it has to be given to another so to exclude other children its not fair. I think it should be made easy for parents to access it. Because other children might have missed it at school, and then they will find it in the clinic. Now if it is not in the clinic, its bad. (HCW6 - Umlazi)

This highlighted a gap in the program: children who miss school-based vaccination have no opportunities for catch-up immunization at clinics or other healthcare facilities.

### Logistical challenges

HCWs identified multiple logistical barriers that hindered program implementation. General logistical difficulties included timely vaccine delivery to all schools:

Sometimes its logistical – getting vaccines to all schools on time can be difficult.

(HCW14 - Wentworth2)

### Transportation and infrastructure barriers

Transportation emerged as a particularly severe barrier, especially for rural communities. One HCW provided a detailed account of the multiple transportation and infrastructure challenges faced:

Sometimes our challenges are about going to the schools. Other areas are not easily accessible. Yes, when it has been raining, we would end up not visiting certain schools because the roads would be damaged therefore not accessible. Some of the other challenges we encounter are transport issues. We would sometimes have to share orders. In the morning you would find that the car would be doing certain things in different facilities then we would have to wait and then we would sometimes arrive late at schools. We cannot properly plan for instance of what we are going to be doing in the morning and which activities to do because the teachers too have their work. We hope to have our own car for school visits. Yes, that would really help us, however, about the road challenges we inform the local councillor to request for tractor to level the roads for us to have access. Sometimes it happens, but on the dates we have booked we sometimes encounter challenges and have to re-book when the time is right. However, we have never had to absolutely cancel our school visit.

(HCW14 - Embo)

## Discussion

### Overview of key findings

This qualitative study examined HCWs and CGs perspectives on the school-based HPV immunization program in eThekwini District in KwaZulu-Natal Province of South Africa, using the WHO BeSD framework. Our findings reveal that HPV vaccine acceptance and uptake in this cervical-cancer high-burden setting are shaped by a complex interplay of factors operating across multiple levels: individual knowledge and emotions, socio-cultural norms and trust dynamics, and crucially, system-level practical barriers. While both HCWs and CGs demonstrated varying levels of awareness about HPV vaccination, the study identified significant knowledge gaps and persistent misinformation that align with broader South African evidence.^20–22^ However, beyond individual-level factors, our findings emphasize that practical access barriers, transportation challenges, infrastructure deficits, and limited vaccine availability beyond school settings can overwhelm individual motivation, necessitating health system strengthening alongside community education efforts.

### Knowledge gaps, misinformation, and the role of HCWs

Despite relatively strong foundational knowledge among HCWs, our study documented concerning awareness gaps among CGs, particularly regarding the link between HPV infection and cervical cancer, and the preventive role of vaccination. This finding is particularly alarming given KwaZulu-Natal’s status as a high-burden region for cervical cancer. The persistence of misinformation, especially myths linking HPV vaccination to infertility, emerged as a critical barrier to vaccine acceptance.^23^ These misconceptions are not unique to eThekwini; similar findings have been reported across South African contexts and internationally, highlighting the need for targeted, evidence-based communication strategies.^21^

Importantly, our study identified HCWs as pivotal influencers in vaccine decision-making. Participants consistently described HCWs as trusted sources of health information, and positive, empathetic communication from HCWs significantly enhanced vaccine acceptance. This finding aligns with international evidence demonstrating that healthcare provider recommendations are among the strongest predictors of HPV vaccine uptake.^24,25^ However, our HCW participants also acknowledged challenges in their practice, including difficulty discussing sexuality-related topics with parents in culturally sensitive ways and insufficient training in addressing vaccine hesitancy. These challenges mirror findings from European studies^26–29^ though South African HCWs face the additional burden of severe resource constraints, language diversity, and deeply entrenched cultural taboos around sexual health discussions.

### Cultural context and the bidirectional role of social factors

Our findings must be understood within KwaZulu-Natal’s unique socio-cultural context, where Zulu traditions significantly influence health behaviors. Sexual health discussions, particularly involving children, remain deeply taboo in many communities, creating barriers to open dialogue about HPV vaccination. This cultural silence provides fertile ground for misinformation to flourish unchallenged. However, our study also revealed that cultural factors can serve as facilitators when appropriately engaged. The cultural value placed on respecting authority figures, including HCWs and community leaders, means that clear, trustworthy recommendations from these sources carry substantial weight. This bidirectional role of culture as both a barrier and potential facilitator represents a unique contribution of our study and differs from many international applications of the BeSD framework that primarily frame culture as an obstacle to overcome.^30,31^

The pronounced rural-urban disparities we observed further contextualize these challenges. Rural participants in areas like Embo faced significantly greater barriers than their urban counterparts in Chatsworth and Umlazi, including limited health infrastructure, transportation challenges, and reduced access to health information.^32^ These geographical disparities contribute directly to the coverage gaps observed in some KwaZulu-Natal sub-districts, where uptake reaches only 40% compared to the national average exceeding 80%.

### System-level barriers: a defining feature in resource-constrained settings

Perhaps the most striking finding from our study is the extent to which practical, system-level barriers can undermine individual motivation and willingness to vaccinate. Even when CGs expressed trust in HCWs and desire to protect their children, tangible obstacles like impassable roads during rainy seasons, lack of dedicated transportation for immunization teams, unavailability of vaccines at primary healthcare facilities for catch-up doses, and consent forms only available in English frequently prevented vaccination. This finding aligns with BeSD framework applications in other low- and middle-income countries (LMICs), where practical barriers often override cognitive and motivational factors.^30,31^

The nature of “missed opportunities” in eThekwini differs fundamentally from those documented in high-income settings. While U.S. studies identify missed opportunities primarily as clinical encounters where vaccination could occur but doesn’t due to provider hesitancy or parental concerns,^33^ in eThekwini, missed opportunities are compounded by genuine access barriers where children may miss school-based vaccination due to absence and then have no accessible alternative. This distinction has profound implications for intervention design: addressing missed opportunities in resource-constrained settings requires substantial infrastructure investment and health system strengthening, not merely improved provider communication.

### Application and utility of the BeSD framework

Our application of the BeSD framework proved highly valuable in capturing the multifactorial nature of vaccine acceptance in this setting. The framework’s four domains – Thinking and Feeling, Social Processes, Motivation, and Practical Issues – effectively organized our understanding of diverse drivers while revealing their dynamic interactions. For instance, we observed that trust in HCWs (Social Processes domain) could partially compensate for limited factual knowledge (Thinking & Feeling domain), enabling vaccine acceptance even among CGs with incomplete understanding of HPV. Conversely, practical barriers (Practical Issues domain) could reinforce community skepticism (Social Processes domain), creating a negative feedback loop that dampens motivation.

The framework’s comprehensive approach prevents overly simplistic attributions of low coverage to single causes and instead illuminates the need for multi-level interventions. Compared to BeSD applications in high-income countries, where confidence and trust often dominate as drivers,^34^ our findings emphasize that in resource-constrained LMIC settings, practical barriers warrant equal if not greater attention. This suggests that the relative importance of BeSD domains varies substantially by context, necessitating locally tailored rather than universal intervention approaches.

### Integration with HIV services: a missed opportunity

An unexpected finding was the limited acknowledgment among participants of the connection between HIV and HPV vaccination, despite KwaZulu-Natal bearing South Africa’s highest HIV burden at 27% prevalence among adults.^35^ Given that HIV-positive women face 3–5 times higher cervical cancer risk due to immunosuppression, this represents a significant missed opportunity for integrated health messaging. Our findings suggest that leveraging existing HIV prevention and treatment platforms which already enjoy substantial community trust and infrastructure could enhance HPV vaccination acceptance and efficiency. This integration strategy has been successful in other contexts and warrants prioritization in KwaZulu-Natal.^26^

### Implications and recommendations

To improve HPV vaccine acceptance and coverage in KwaZulu-Natal, several actionable strategies are proposed:

1. Culturally adapted communication: Develop multilingual, visually oriented materials in isiZulu and other local languages to address varying literacy levels.
2. Enhanced HCW training: Build capacity for culturally competent communication and empathetic counseling to address hesitancy effectively.
3. System-level investments: Prioritize transportation, cold-chain logistics, and consistent vaccine availability at clinics beyond schools.
4. Community engagement: Involve trusted influencers i.e. teachers, traditional leaders, and faith figures as vaccination champions.
5. Service integration: Incorporate HPV vaccination within HIV prevention and treatment programs to maximize reach and efficiency.

## Strengths and limitations

Our study’s strengths include the use of a robust theoretical framework (WHO BeSD), diverse maximum variation sampling across urban-rural settings, rigorous analytical processes with independent coding, and contextually relevant findings that directly inform local policy needs.

The study utilizes qualitative methods to offer a nuanced understanding of the HCWs and CGs’ knowledge, perceptions, and experiences regarding the school-based HPV immunization program in eThekwini district. Its contextual relevance is heightened by its localized focus, but its generalizability may be limited, and the study’s findings could be impacted by data interpretation.

Furthermore, the study’s exclusive focus on healthcare workers and caregivers may overlook other crucial viewpoints, and ethical considerations must be carefully addressed. The predominantly female, school health nurse composition of our HCW sample limits generalizability to other healthcare settings and cadres.

Important stakeholder perspectives particularly adolescents themselves, teachers, traditional leaders, and policymakers were not captured. Social desirability bias may have influenced self-reported attitudes, and translation from isiZulu to English may have resulted in some loss of cultural nuance. The cross-sectional design prevented understanding of how perspectives evolve over time as the program matures and communities gain familiarity with vaccination. Nonetheless, the study contributes to policy and practice aimed at bettering HPV immunization programs, emphasizing the need for broader stakeholder involvement and rigorous methodology in future studies.

## Conclusion

This study demonstrates that improving HPV vaccine acceptance and uptake in eThekwini District requires comprehensive, multi-level interventions that simultaneously address knowledge deficits, leverage trusted community relationships, engage cultural values as facilitators rather than viewing them solely as barriers, and critically, dismantle the practical system-level obstacles that prevent access even among motivated populations. The WHO BeSD framework proved valuable in revealing these complex, interacting drivers and should guide future intervention design. While our findings reflect the unique context of KwaZulu-Natal, they offer important lessons for similar high-burden, resource-constrained settings throughout South Africa and sub-Saharan Africa. Ultimately, achieving high HPV vaccination coverage in such contexts demands not only individual-level education but equally, substantial investment in health system strengthening, infrastructure development, and culturally grounded community engagement strategies.

## What is already know on this topic

Human papillomavirus (HPV) is one of the most common sexually transmitted diseases (STIs) globally. HPV is known to cause various cancers, including cervical cancer, and HPV-related cancers have a significant global burden

## What this study adds

This research study indicates that taking proactive measures by involving the community is crucial to tackling misinformation.

The need for extensive training and education programs is clear, since (some) HCWs and CGs have low understanding and awareness regarding the HPV vaccine.

## References

[1] Bruni L, Saura-Lázaro A, Montoliu A, Brotons M, Alemany L, Diallo MS, Afsar OZ, LaMontagne DS, Mosina L, Contreras M, et al. HPV vaccination introduction worldwide and WHO and UNICEF estimates of national HPV immunization coverage 2010–2019. Prev Med. 2021;144:106399. 33388322 10.1016/j.ypmed.2020.106399

[2] Arbyn M, Weiderpass E, Bruni L, de Sanjosé S, Saraiya M, Ferlay J, Bray F. Estimates of incidence and mortality of cervical cancer in 2018: a worldwide analysis. Lancet Glob Health. 2020;8(2):e191–14. doi: 10.1016/S2214-109X(19)30482-6. 31812369 PMC7025157

[3] Amani A, Bloem P, Kobayashi E, Atuhaire B, Wiysonge CS, Impouma B. Scaling HPV vaccination in Africa to eliminate cervical cancer by 2030. Lancet Glob Health. 2025;13(12):S2214–109X(25)00349–3. 10.1016/S2214-109X(25)00349-3 41106416

[4] Padarath A, Moeti TL. South African health review 2022: Health systems recovery after COVID-19. S Afr Health Rev. 2023;25. doi: 10.61473/001c.87567.

[5] Petrosky E, Bocchini Jr JA, Hariri S, Chesson H, Curtis CR, Saraiya M, Unger ER, Markowitz LE, Centers for Disease Control and Prevention (CDC). Use of 9-valent human papillomavirus (HPV) vaccine: updated HPV vaccination recommendations of the Advisory Committee on Immunization Practices. MMWR Morb Mortal Wkly Rep. 2015;64(11):300–304. 25811679 PMC4584883

[6] Koulova A, Tsui J, Irwin K, Van Damme P, Biellik R, Aguado MT. Country recommendations on the inclusion of HPV vaccines in national immunization programmes among high-income countries, June 2006–January 2008. Vaccine. 2008;26(51):6529–6541. 18805453 10.1016/j.vaccine.2008.08.067

[7] Small W Jr, Bacon MA, Bajaj A, Chuang LT, Fisher BJ, Harkenrider MM, Jhingran A, Kitchener HC, Mileshkin LR, Viswanathan AN, et al. Cervical cancer: a global health crisis. Cancer. 2017;123(13):2404–2412. 28464289 10.1002/cncr.30667

[8] Bray F, Ferlay J, Soerjomataram I, Siegel RL, Torre LA, Jemal A. Global cancer statistics 2018: GLOBOCAN estimates of incidence and mortality worldwide for 36 cancers in 185 countries. Cancer J Clin. 2018;68(6):394–424. doi: 10.3322/caac.21492. 30207593

[9] Delany-Moretlwe S, Kelley KF, James S, Scorgie F, Subedar H, Dlamini NR, Pillay Y, Naidoo N, Chikandiwa A, Rees H. Human papillomavirus vaccine introduction in South Africa: implementation lessons from an evaluation of the national school-based vaccination campaign. Glob Health Sci Pract. 2018;6(3):428–438. doi: 10.9745/GHSP-D-18-00090. PMC6172125 30143561

[10] Butt M, Mohammed R, Butt E, Butt S, Xiang J. Why have immunization efforts in Pakistan failed to achieve global standards of vaccination uptake and infectious disease control? Risk Manag Healthcare Policy. 2020;12:111–124. 10.2147/RMHP.S211170 PMC7024803 32104117

[11] Hilton S, Hunt K, Bedford H, Petticrew M. School nurses’ experiences of delivering the UK HPV vaccination programme in its first year. BMC Infect Dis. 2011;24(1):226. doi: 10.1186/1471-2334-11-226. PMC3176210 21864404

[12] Wiysonge CS, Paulsen E, Lewin S, Ciapponi A, Herrera CA, Opiyo N, Pantoja T, Rada G, Oxman AD. Financial arrangements for health systems in low‐income countries: an overview of systematic reviews. Cochrane Database Syst Rev. 2017;2017(9). doi: 10.1002/14651858.CD011084.pub2. PMC5618470 28891235

[13] Amponsah-Dacosta E, Blose N, Nkwinika VV, Chepkurui V. Human papillomavirus vaccination in South Africa: programmatic challenges and opportunities for integration with other adolescent health services? Front Public Health. 2022;10:799984. doi: 10.3389/fpubh.2022.799984. 35174123 PMC8841655

[14] WHO. Understanding the behavioural and social drivers of vaccine uptake. WHO position paper - May 2022. Wkly Epidemiol Rec. 2022;97(20):209–224.

[15] Williams H. The meaning of “phenomenology”: qualitative and philosophical phenomenological research methods. Qual Rep. 2021;26(2):366–385. doi: 10.46743/2160-3715/2021.4587.

[16] Renjith V, Yesodharan R, Noronha JA, Ladd E, George A. Qualitative methods in health care research. Int J Preventative Med. 2021;12(1). doi: 10.4103/ijpvm.IJPVM_321_19. PMC8106287 34084317

[17] Patton M. Qualitative research & evaluation methods. Thousand Oaks (CA): SAGE Publications; 2001.

[18] Bhengu TT, Mbokazi SS. Reflections on doing a participatory action research: case study of three rural communities in KwaZulu-Natal. Stud Tribes Tribals. 2015;13(2):172–180. doi: 10.1080/0972639X.2015.11886724.

[19] Braun V, Clarke V. Using thematic analysis in psychology. Qual Res Phychol. 2006;3(2):77–101. doi: 10.1191/1478088706qp063oa.

[20] Ngcobo NJ, Burnett RJ, Cooper S, Wiysonge CS. Human papillomavirus vaccination acceptance and hesitancy in South Africa: research and policy agenda. S Afr Med J. 2019;109(1):13–15. 10.7196/SAMJ.2018.v109i1.13723 30606297

[21] Milondzo T, Meyer JC, Dochez C, Burnett RJ. Misinformation drives low human papillomavirus vaccination coverage in South African girls attending private scho Milondzo, Tracy, et al. “Misinformation drives low human papillomavirus vaccination coverage in South African girls attending private schools.” Frontiers in Public Health 9 (2021): 598625. Front Public Health. 2021;9:598625. 33681125 10.3389/fpubh.2021.598625 PMC7933005

[22] Moodley S. Defining city-to-city learning in Southern Africa: exploring practitioner sensitivities in the knowledge transfer process. Habitat Int. 2019;85:34–40.

[23] Ortiz RR, Smith A, Coyne-Beasley T. A systematic literature review to examine the potential for social media to impact HPV vaccine uptake and awareness, knowledge, and attitudes about HPV and HPV vaccination. Hum Vacc Immunother. 2019;15(7–8):1465–1475. doi: 10.1080/21645515.2019.1581543. PMC6746532 30779682

[24] Soon R, Dela Cruz MR, Tsark JU, Chen JJ, Braun KL. A survey of physicians’ attitudes and practices about the human papillomavirus (HPV) vaccine in Hawaiʻi. Hawaiʻi J Med Public Health. 2015;74(7):234–241. PMC4507363 26225269

[25] Bakare D, Gobbo E, Akinsola KO, Bakare AA, Salako J, Hanson C, Herzig van Wees S, Falade A, King C. Healthcare worker practices for HPV vaccine recommendation: a systematic review and meta-analysis. Hum Vacc Immunother. 2024;20(1):2402122. doi: 10.1080/21645515.2024.2402122. PMC11486212 39400296

[26] Karafillakis E, Simas C, Jarrett C, Verger P, Peretti-Watel P, Larson HJ. HPV vaccination in Europe: drivers of coverage, barriers, and enablers. Vaccine. 2019;37(2):219–229.28760612

[27] Somayaji KH. Vaccination hesitancy through the ages: the past, present, and future implications. Temple University; 2023.

[28] Napolitano F, Pelullo CP, Della Polla G, Angelillo IF. HPV vaccination attitudes and behaviors among general practitioners in Italy. Vaccine. 2021;9(1):63. doi: 10.3390/vaccines9010063. PMC7832300 33477779

[29] Gobert C, Semaille P, Van der Schueren T, Verger P, Dauby N. Prevalence and determinants of vaccine hesitancy and vaccines recommendation discrepancies among general practitioners in French-speaking parts of Belgium. Vaccines. 2019;9(7):771 doi:10.3390/vaccines9070771. PMC8310255 34358187

[30] Vandecasteele R, Robijn L, Willems S, De Maesschalck S, Stevens PAJ. Barriers and facilitators to culturally sensitive care in general practice: a reflexive thematic analysis. BMC Primary Care. 2024;25(1):381. doi: 10.1186/s12875-024-02630-y. 39443846 PMC11515484

[31] Zhang E, Shang S, Xing Y, Cui J, Pan C, Seale H, Fang Q. Mapping behavioral and social drivers of influenza vaccine uptake in older adults: a scoping review. Vaccine. 2025;13(6):624. doi: 10.3390/vaccines13060624. PMC12197636 40573955

[32] Moshabela M, Bukenya D, Darong G, Wamoyi J, McLean E, Skovdal M, Ddaaki W, Ondeng’e K, Bonnington O, Seeley J, et al. Traditional healers, faith healers and medical practitioners: the contribution of medical pluralism to bottlenecks along the cascade of care for HIV/AIDS in Eastern and Southern Africa. Sex Transm Infect. 2017;93(3 Suppl):e052974. doi: 10.1136/sextrans-2016-052974. 28736393 PMC5739844

[33] Shay LA, Baldwin AS, Betts AC, Marks EG, Higashi RT, Street RL Jr, Persaud D, Tiro JA. Parent-provider communication of HPV vaccine hesitancy. Pediatrics. 2018;141(6):e20172312. doi: 10.1542/peds.2017-2312. 29765009 PMC6005174

[34] Larson HJ. Defining and measuring vaccine hesitancy. Nat Hum Behav. 2022;6(12):1609–1610. doi: 10.1038/s41562-022-01484-7. 36418535 PMC9684976

[35] Human Sciences Research Council. Annual report 2017/18. Pretoria: Human Sciences Research Council; 2018.

